# LncRNA DUXAP9‐206 directly binds with Cbl‐b to augment EGFR signaling and promotes non‐small cell lung cancer progression

**DOI:** 10.1111/jcmm.14085

**Published:** 2018-12-04

**Authors:** Ting Zhu, Shu An, Man‐Ting Choy, Junhao Zhou, Shanshan Wu, Shihua Liu, Bangdong Liu, Zhicheng Yao, Xun Zhu, Jueheng Wu, Zhenjian He

**Affiliations:** ^1^ School of Public Health Sun Yat‐sen University Guangzhou China; ^2^ Department of Microbiology Zhongshan School of Medicine Sun Yat‐sen University Guangzhou China; ^3^ Department of Laboratory Medicine Cancer Center of Guangzhou Medical University Guangzhou Guangdong China; ^4^ Key Laboratory of Tropical Disease Control (Sun Yat‐sen University) Ministry of Education Guangzhou China; ^5^ Department of Endocrinology The First Affiliated Hospital of Sun Yat‐sen University Guangzhou Guangdong China; ^6^ Department of General Surgery The Third Affiliated Hospital of Sun Yat‐sen University Guangzhou Guangdong China; ^7^ Guangdong Provincial Key Laboratory of Orthopedics and Traumatology Guangzhou China

**Keywords:** Cbl‐b, DUXAP9‐206, EGFR signaling, lncRNA, non‐small cell lung cancer

## Abstract

Long noncoding RNAs (lncRNAs) are involved in the pathology of various tumours, including non‐small cell lung cancer (NSCLC). However, the underlying molecular mechanisms of their specific association with NSCLC have not been fully elucidated. Here, we report that a cytoplasmic lncRNA, DUXAP9‐206 is overexpressed in NSCLC cells and closely related to NSCLC clinical features and poor patient survival. We reveal that DUXAP9‐206 induced NSCLC cell proliferation and metastasis by directly interacting with Cbl‐b, an E3 ubiquitin ligase, and reducing the degradation of epidermal growth factor receptor (EGFR) and thereby augmenting EGFR signaling in NSCLC. Notably, correlations between DUXAP9‐206 and activated EGFR signaling were also validated in NSCLC patient specimens. Collectively, our findings reveal the novel molecular mechanisms of DUXAP9‐206 in mediating the progression of NSCLC and DUXAP9‐206 may serve as a potential target for NSCLC therapy.

## 1 INTRODUCTION

Lung cancer is a leading cause of cancer‐related mortality worldwide.^1^, ^2^ Non‐small cell lung cancer (NSCLC), which includes several histological subtypes, such as lung adenocarcinoma, lung squamous cell carcinoma, large‐cell lung carcinoma and several other histologic types accounts for over 85% of all lung cancer cases.^2^ Although significant progress has been made in current therapeutic strategies, the overall 5‐year survival rate is still only 15% with all stages and subtypes combined and the prognosis for majority of the patients remains poor.^3^ Thus, exploration of the molecular mechanisms and molecules involved in the development and progression of NSCLC may provide valuable therapeutic targets for these diseases.

It has been shown that protein‐coding genes occupy only a small proportion (1%‐2%) of the human genome. The rest are non‐protein‐coding transcripts including small noncoding RNAs and long noncoding RNAs (lncRNAs).^4^ LncRNAs have gradually been shown to be pivotal molecules that affect cancer development and progression.^5^ The length of lncRNAs is longer than 200 nucleotides (nt), yet this class of RNA has limited coding potential.^6^ LncRNAs function in a wide range of biological processes and can regulate gene expression through diverse mechanisms.^7^ It has been suggested that cytoplasmic lncRNAs are important molecules in regulating cancer intracellular signaling pathways.^8^ For example, the lncRNA MAYA modulates the methylation status of MST1 leading to bone metastasis.^9^ The lncRNA lncAKHE enhances cell growth and migration in hepatocellular carcinoma via activation of NOTCH2 signaling.^10^

Epidermal growth factor receptor (EGFR), a transmembrane glycoprotein with tyrosine kinase activity is often aberrantly activated by mutation or overexpression in many human cancers.^11^ Over 60% of the NSCLC cases show EGFR overexpression, which is associated with poor prognosis of NSCLC.^12^, ^13^ Ligand‐activated EGFR leads to stimulation of intracellular cascades, such as the RAS/RAF/ERK and PI3K/AKT signaling pathways. Abnormally activated EGFR drives the malignant phenotype including cell proliferation, survival, invasion and migration.^13^ Although several lncRNAs have been reported to modulate tumour proliferation, apoptosis or metastasis, the specific mechanisms of lncRNA involvement in EGFR signaling pathway‐mediated tumour progression of NSCLC are unclear. Here, we found that a cytoplasmic lncRNA, DUXAP9‐206 is overexpressed in NSCLC cells and closely related to NSCLC clinical features and poor patient survival. Furthermore, we demonstrated that DUXAP9‐206 induced NSCLC cell proliferation and metastasis by directly interacting with Cbl‐b, an E3 ubiquitin ligase and reducing the degradation of EGFR, thus activating the EGFR signaling pathway. Overall, our findings reveal the novel molecular mechanisms of DUXAP9‐206 in mediating the progression of NSCLC and DUXAP9‐206 may serve as a potential target for NSCLC therapy.

## 2 MATERIALS AND METHODS

### 2.1 Cell culture

NSCLC cell lines including 95D, A549, Calu‐1, H1299, H1650, NCI‐H1703, NCI‐H1975, NCI‐H2009, NCI‐H2030, NCI‐H460, HCC827, PC9 and SK‐MES‐1 were purchased from ATCC and maintained in Dulbecco's modified Eagle's medium (DMEM) supplemented with 10% fetal bovine serum (Corning, Corning, NY, USA). The BEAS‐2B immortalized human bronchial epithelial cell line was purchased from the Shanghai Institutes of Biological Sciences (Shanghai, China) and cultured according to the instructions of the manufacturer.

### 2.2 Tissue specimens

A total of 216 paraffin‐embedded NSCLC specimens, were clinically and histopathologically diagnosed at the Sun Yat‐sen University Cancer Center. These specimens are defined as the SYSUCC cohort. NSCLC tissue and paired adjacent non‐cancerous lung tissue specimens were stored frozen in liquid nitrogen until further use. Adjacent non‐tumour tissue specimens were taken a standard distance (3 cm) from the margin of resected tissues of patients with NSCLC who underwent surgical lung resection and confirmed by pathological diagnosis. For the use of these clinical materials for research purposes, prior patient consents and approval from the Institutional Research Ethics Committee of the Sun Yat‐sen University were obtained. Clinical information of the samples is described in detail in Table S1.

### 2.3 Plasmids and generation of stable cell lines

Full‐length DUXAP9‐206 was synthesized by Genewiz (Suzhou, China) and was inserted into the pSin‐EF2‐puro retroviral vector (Addgene, Cambridge, MA, USA). DUXAP9‐206 specific short hairpin RNA (shRNA) oligonucleotides were annealed and then ligated into the pSuper‐retro‐puro plasmid to generate pSuper‐DUXAP9‐206‐RNAi#1 and pSuper‐DUXAP9‐206‐RNAi#2. pCMV6‐Entry‐EGFR and pCMV6‐Entry‐Cbl‐b were purchased from Origene Technologies (Rockville, MD, USA). Co‐transfection with the packaging plasmid in 293FT cells was performed with a standard calcium phosphate transfection method as previously described.^14^ Briefly, the cells were transfected for 24 hours and supernatants were collected and incubated with the indicated cells with polybrene (2.5 μg/mL). Positive cells were selected with puromycin (1.5 μg/mL) for 10 days.

### 2.4 Small interfering RNAs

All siRNAs were synthesized by RiboBio (Guangzhou, China) and used at a final concentration of 100 nmol/L. The sequences of the siRNAs are summarized in Table S3.

### 2.5 5′ and 3′ rapid amplification of cDNA ends PCR

Total RNA extracted from human A549 and H1703 cells was subjected rapid amplification of cDNA ends polymerase chain reaction (RACE PCR) (Ambion, Austin, TX, USA) according to the manufacturer's specifications. Gene‐specific primers used for the PCR of RACE analysis are listed in Table S4.

### 2.6 Cell nucleus/cytoplasm fraction isolation

Cell nucleus/cytoplasm fraction isolation was performed using Nuclear and Cytoplasmic Extraction Kit (Ambion). In brief, cells were washed with ice‐cold PBS two times and then ice‐cold CERI, CERII and NER reagents were added sequentially. After vortex and short centrifugation, the supernatant was collected as cytoplasmic fraction and the remainder with additional washing was considered as nuclear pellets.

### 2.7 Western blotting analysis

Western blotting analysis was performed according to a previously described standard method^15^ using the antibodies anti‐Cbl‐b (ab93205, 1:500; Abcam, Cambridge, MA, USA), anti‐EGFR (ab52894, 1:1000; Abcam), anti‐Flag (F2555, 1:2000; Sigma, St Louis, MO, USA), anti‐ubiquitin (ab7780, 1:1000; Abcam), anti‐p‐AKT (ab81283, 1:500; Abcam), anti‐AKT (ab8805, 1:1000; Abcam), anti‐p‐ERK (4370, 1:500; Cell Signaling, Danvers, MA, USA) and anti‐ERK (4695, 1:1000; Cell Signaling). The blotted membranes were stripped and re‐blotted with anti‐actin (ab8227, 1:2000; Abcam) as a loading control.

### 2.8 RNA extraction and quantitative reverse transcription‐PCR

Total RNA of cultured cells or clinical NSCLC specimens was extracted using TRIzol (Invitrogen, San Diego, CA) according to the manufacturer's instructions. We first measured the quantity of mRNA as described previously.^16^ First‐strand cDNA was generated by MMLV transcriptase (Promega, Madison, WI). Quantitative reverse transcription (qRT)‐PCR was performed on a CFX96 qRT‐PCR detection system (Bio‐Rad, Richmond, CA, USA). The sequences of the primers are listed in Table S5.

### 2.9 Co‐immunoprecipitation

The indicated cells were collected and lysed with cell lysis buffer (25 mmol/L HEPES, 150 mmol/L NaCl, 1 mmol/L EDTA, 1% NP‐40, 2% glycerol, 0.2% cocktail, pH 7.4) supplemented with protease inhibitor cocktail (Promega) and then, the lysate was incubated with magnetic beads coated with EGFR antibodies or anti‐mouse immunoglobulin G (IgG) agarose beads (Sigma) that were washed six times in buffer containing 20 mmol/L HEPES, 300 mmol/L NaCl, 1 mmol/L EDTA, 0.1% NP‐40 and 0.1% glycerol, pH 7.4. Western blotting analysis was used to detect both the input and co‐immunoprecipitation (Co‐IP) protein lysis.

### 2.10 RNA immunoprecipitation

RNA immunoprecipitation (RIP) was performed using a Magna RIP RNA‐Binding Protein Immunoprecipitation Kit (Millipore, Billerica, MA, USA) according to the manufacturer's instructions. The indicated cells were lysed in the provided buffer and immunoprecipitated with the indicated antibody or control IgG (Abcam). Extracted RNA was then subjected to qRT‐PCR analysis.

### 2.11 RNA pull‐down assay

A T7 High Yield RNA Synthesis Kit (Ambion) was used to obtain in vitro transcribed RNA according to the manufacturer's instructions. The transcribed RNA was labelled using a 3′ End Biotinylation Kit (Thermo) and the protein that interacted with the RNA was enriched with an RNA‐Protein Pull‐Down kit (Thermo) according to the manufacturer's instructions. In brief, biotinylated labelled RNA was incubated with washed streptavidin beads at 4°C. Then, cell lysate was added to the binding reaction and further incubated overnight. Retrieved proteins were detected by Western blotting analysis or mass spectrometry (MS) identification.

### 2.12 Wound healing and transwell assays

For wound healing assays, the indicated cells were plated in 6‐well plates and then, streaks were created with a pipette tip. After scratching, wells were gently washed with PBS twice and incubated in DMEM supplemented with 1% FBS. Progression of migration was observed and photographed at 24 hours after wounding.

Cell invasion and migration assays were performed using a 24‐well Transwell chamber (8 μm; Corning) with or without coated Matrigel (BD Biosciences). The upper chamber of the Transwell device was filled with serum‐free DMEM and the lower chamber of the Transwell device was filled with DMEM supplemented with 10% FBS. After incubation for 24 hours, cells invading the bottom side of the inserts were fixed with methanol stained with 0.1% crystal violet (Sigma), photographed and quantified under a microscope (Carl Zeiss, Oberkochen, Germany) by counting them in 5 random fields.

### 2.13 Cell proliferation assay

For colony formation assays, the indicated cells were plated in six‐well plates (5 × 10^2^ cells) and cultured for 10 days. Then, the cells were fixed with 4% formaldehyde for 15 minutes and stained with 0.1% crystal violet for 10 minutes.

For 3‐(4,5‐dimethyl‐2‐thiazolyl)‐2,5‐diphenyl‐2H‐tetrazolium bromide (MTT) assays, a total of 1 × 10^3^ cells were seeded in 96‐well plates, stained with 100 mL of sterile MTT dye (0.5 mg/mL, Sigma) for 4 hours at 37°C, followed by removal of the culture medium with addition of 150 mL of dimethyl sulfoxide (Sigma). The samples were shaken at room temperature for 10 minutes and then, the absorbance of the stained cells was measured at 570 nm, with 655 nm as the reference wavelength.

### 2.14 Tumour xenografts and metastasis models

All experimental procedures were approved by the Institutional Animal Care and Use Committee of Sun Yat‐sen University. For tumour growth assays, cells (3 × 10^6^) were injected into the inguinal folds of the BALB/c nude mice (6‐7 weeks of age, 17‐20 g, female). After 35 days, the animals were killed and tumours were removed, weighed and sectioned. For metastasis assays, cells (1 × 10^6^) were injected into the tail vein of age‐matched BALB/c nude mice. Bioluminescent imaging was performed using the Xenogen IVIS Spectrum Imaging System (Caliper Life Sciences, Hopkinton, MA, USA) with administration of D‐luciferin (150 mg/kg i.v.) and anaesthesia by isoflurane. Sixty days after tumour implantation, the mice were killed and the lungs were collected to count surface metastases. Extracted lungs were fixed in formalin and embedded in paraffin using a conventional method for further histological hematoxylin and eosin (H&E) staining.

### 2.15 In situ hybridization and immunohistochemistry

Formalin‐fixed, paraffin‐embedded NSCLC samples were fixed in 4% paraformaldehyde and incubated with proteinase K for 20 minutes at 37°C. After they were dewaxed and rehydrated, the samples were hybridized with 40 nmol/L digoxin‐labelled DUXAP9‐206 probe (Exiqon, for sequence, see Table S6) at 55°C overnight. The slides were then washed with SSC buffer and incubated with anti‐digoxin monoclonal antibody (Roche) for 1 hour at room temperature followed by staining with Nuclear Fast Red solution.

Immunohistochemistry assays were performed and quantified as previously described^17^ using the following primary antibodies: anti‐EGFR (1:100; Abcam), anti‐p‐AKT (1:50; Abcam) and anti‐p‐ERK (1:50; Abcam). The staining scores were evaluated by both the staining intensity and proportion of positively stained tumour cells. Scores representing the proportion of positively stained tumour cells were as follows: 0, no positive cells; 1, <10%; 2, 10%‐50%; and 3, >50%. The staining intensity was determined as follows: (no staining), 1 (week staining), 2 (moderate staining) and 3 (strong staining). The staining index (SI) was calculated as the product of staining intensity × proportion of positively stained cells resulting in scores of 0, 1, 2, 3, 4, 6 or 9. An SI score of 3 was used as a cut‐off value based on heterogeneity measurement using the log‐rank test with respect to overall survival for the expression of DUXAP9‐206 or the protein of interest.

### 2.16 Statistical analysis

All statistical analyses were carried out using the SPSS 20.0 statistical software package. Survival curves were analysed by the Kaplan‐Meier method and a log‐rank test was used to assess significance. The correlation between the expression levels of DUXAP9‐206 and clinical parameters of patients was assayed by a chi‐squared test. Student's *t* test was used to compare between groups. In all cases, error bars represent the mean ± SD derived from three independent experiments. *P* values <0.05 were considered statistically significant.

## 3 RESULTS

### 3.1 DUXAP9‐206 is up‐regulated in NSCLC and correlates with patients’ survival

**Figure 1:**
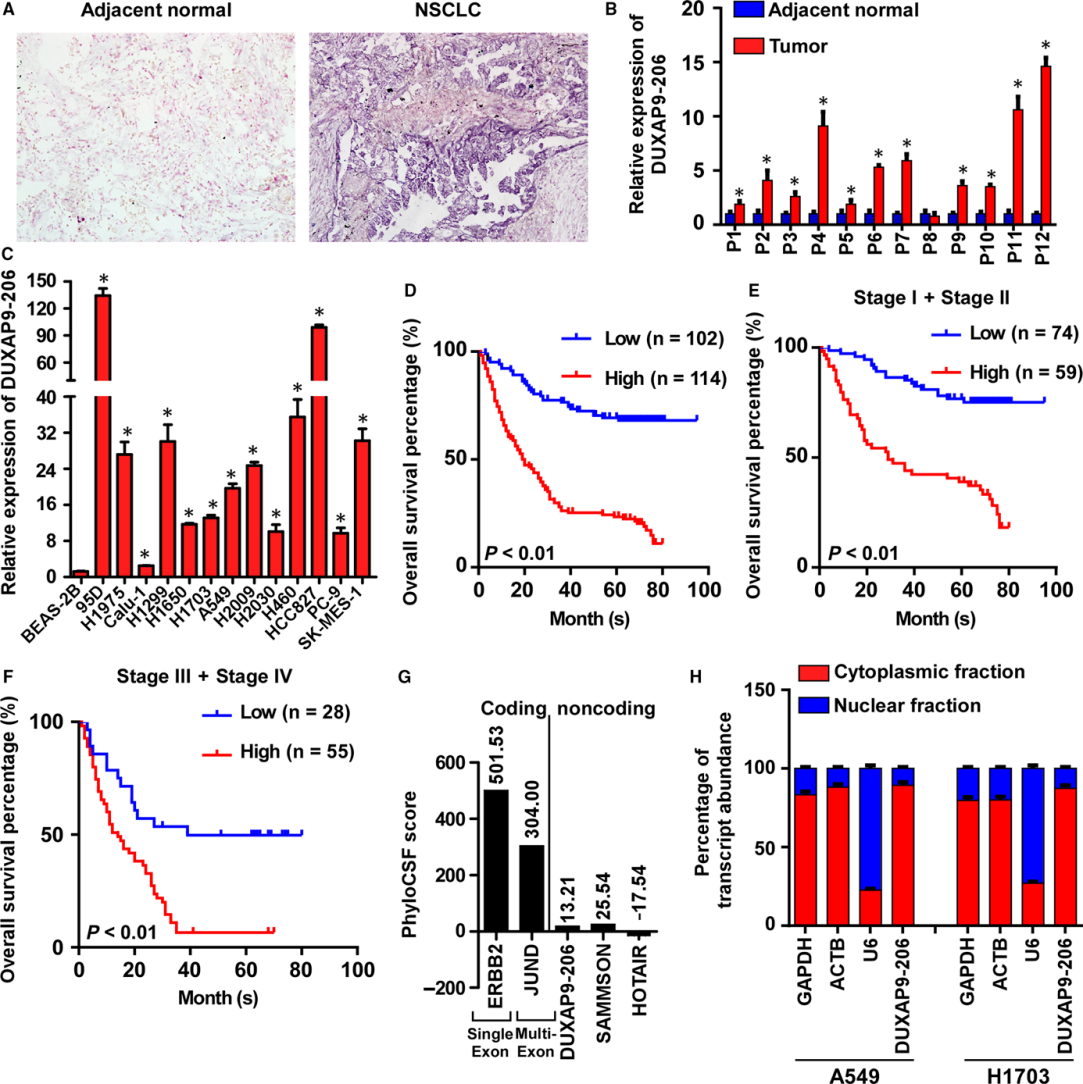
DUXAP9‐206 is highly expressed in NSCLC and correlated with poor prognosis. (A) DUXAP9‐206 is up‐regulated in NSCLC tumour specimens as detected by ISH assays. (B) Relative expression of DUXAP9‐206 in NSCLC tumour tissues and corresponding adjacent non‐tumourous lung tissues by qRT‐PCR. *P < 0.05. (C) Analysis of DUXAP9‐206 expression in NSCLC cell lines and the immortalized human bronchial epithelial cell line BEAS‐2B. *P < 0.05. (D‐F) Kaplan‐Meier survival analysis of the correlation between the DUXAP9‐206 expression and overall survival in SYSUCC NSCLC cohorts (D‐F). (G) The maximum CSF scores of DUXAP9‐206 and other known coding and noncoding RNAs (served as control) were assessed by PhyloCSF analysis. (H) qRT‐PCR analysis of DUXAP9‐206 and other known nuclear or cytoplasmic RNA molecules (served as positive controls) in nuclear and cytoplasmic fractions

To explore the functional and clinical relevance of DUXAP9‐206 in NSCLC, we initially analysed the expression levels of DUXAP9‐206. In situ hybridization (ISH) analysis using a specific probe revealed that the expression levels of DUXAP9‐206 were significantly elevated in NSCLC tumour tissues compared with paired adjacent non‐tumour tissues (Figure 1A). The level of DUXAP9‐206 in NSCLC tissues was further verified by qRT‐PCR assays with specific primer (Figure 1B). Consistently, DUXAP9‐206 was highly expressed in NSCLC cell lines compared with normal lung epithelial cells (BEAS‐2B) (Figure 1C). Moreover, in a set of 216 NSCLC patients in the Sun Yat‐sen University Cancer Center (SYSUCC) cohort (Table S1) for whom overall survival data were available, patients with high DUXAP9‐206 expression (SI >3) had shorter overall survival times than those with low DUXAP9‐206 (SI ≤3) expression suggesting that DUXAP9‐206 level might be indicative of the prognosis of NSCLC (Figure 1D‐F). Moreover, early‐stage (stages I and II together) and late‐stage (stages III and IV together) patients with high DUXAP9‐206 level had shorter survival times than those with low DUXAP9‐206 expression implying that DUXAP9‐206 level might be indicative of the prognosis of NSCLC patients at various clinical stages. Furthermore, through analysis of the correlation between DUXAP9‐206 level and clinicopathologic features of NSCLC patients from the SYSUCC cohort, we found that DUXAP9‐206 expression was significantly associated with T classification, LN metastasis and clinical stage demonstrating its relationship to tumour progression (Table S2).

The full‐length sequence of lncRNA DUXAP9‐206 was obtained by RACE PCR. The results showed that DUXAP9‐206, located on human chromosome 14 (Chromosome 14: 19 062 316‐19 115 270) (http://www.ensembl.org/index.html), is composed of 1426 nucleotides (Figure S1A‐C). Similar to the structure of mRNA, DUXAP9‐206 possesses a 5′‐terminal cap and a 3′‐terminal poly (A) structure (Figure S1D,E). Analysis of the sequences by coding potential calculator software indicated that DUXAP9‐206 failed to encode a protein and has no conserved domain (Figure S1F). Moreover, a codon substitution frequency (CSF) analysis using PhyloCSF, an algorithm based on evolutionary signatures, showed that DUXAP9‐206 had a low CSF score (13.21) compared with other well‐characterized lncRNAs (eg SAMMSON: 25.54; and HOTAIR: 17.54) and coding RNAs (eg ERBB2: 501.53 a; and JUND: 304.00), which further indicated that DUXAP9‐206 lacks protein‐coding potential (Figure 1G). Using RT‐PCR of nuclear and cytoplasmic fractions, we found that DUXAP9‐206 was mainly located in the cytoplasm of NSCLC cells (Figure 1H).

### 3.2 DUXAP9‐206 promotes a proliferative and prometastatic phenotype

**Figure 2:**
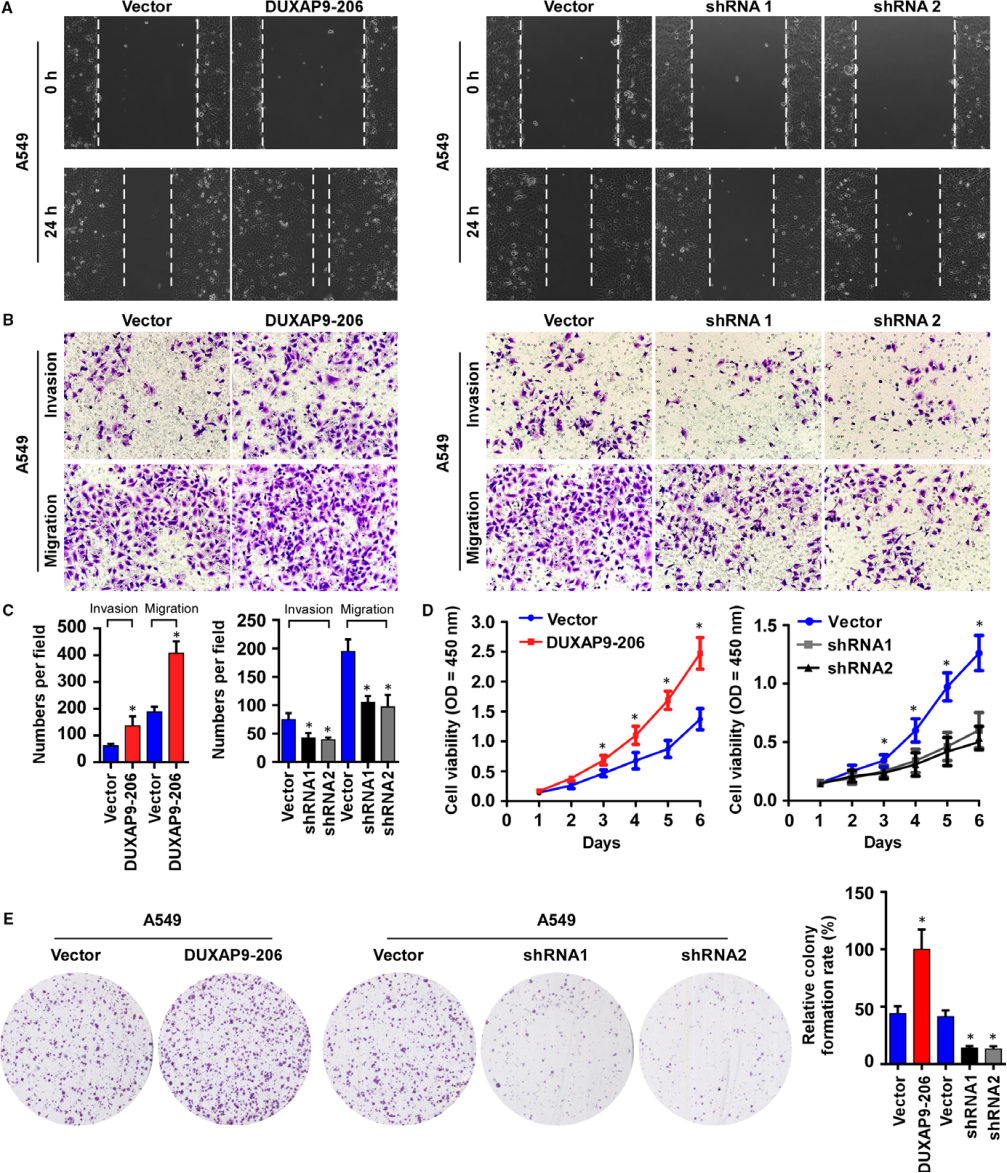
DUXAP9‐206 promotes NSCLC cell proliferation and invasion in vitro. (A) Representative micrographs of wound closures at 0 and 24 h after wounding. (B) The indicated invading or migrating cells analysed by Matrigel‐coated or noncoated Transwell assays respectively. (C) Quantification of the indicated invading or migrating cells in 5 random fields analysed by Transwell assays. *P < 0.05. (D) MTT assays were performed in the indicated cells. (E) Representative micrographs (left panel) and quantification (right panel) of colony formation. *P < 0.05

To further understand the biological effect of DUXAP9‐206 deregulation in vitro*,* we established NSCLC cell lines (A549 and H1703) with stable overexpression or knockdown of DUXAP9‐206 (Figure S2A,B). The results of wound healing assays showed that DUXAP9‐206 overexpression increased the migratory speed and that depletion of DUXAP9‐206 decreased the cell migration in both A549 and H1703 cells (Figure 2A and Figure S3A). Moreover, DUXAP9‐206 overexpression significantly enhanced the invasiveness and migration of the A549 and H1703 cells with Matrigel‐coated or uncoated Transwell assays, whereas inhibition of DUXAP9‐206 markedly weakened the cellular invasive and migratory capabilities (Figure 2B,C and Figure S3B,C). Furthermore, our results showed that overexpression of DUXAP9‐206 remarkably increased the cell growth rate and colony formation abilities, whereas knockdown of DUXAP9‐206 impaired these abilities in both A549 and H1703 cells (Figure 2D,E and Figure S3D,E). Collectively, our data suggest that DUXAP9‐206 greatly contributes to the proliferation, invasion and migration of NSCLC cells in vitro.

### 3.3 DUXAP9‐206 enhances metastasis and proliferation

**Figure 3:**
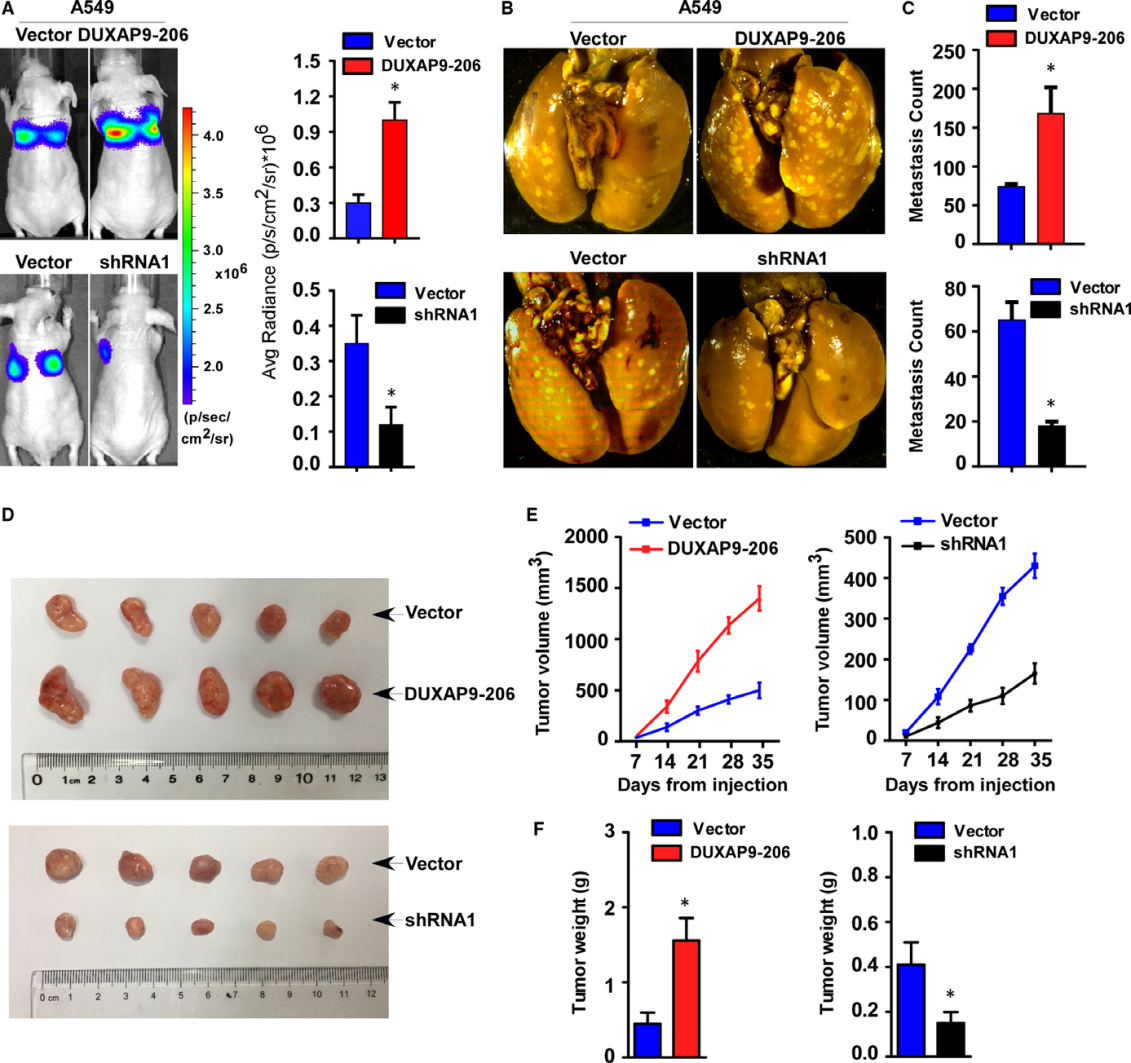
DUXAP9‐206 enhances proliferation and metastasis of NSCLC in vivo. (A) Representative bioluminescence images of lung metastases in the mice that received tail vein injections of the indicated cells. Quantification of bioluminescent signal. *P < 0.05. (B) Representative bright‐field images of the lungs. After 8 weeks, mice were anaesthetized and lung tissue specimens were collected. (C) Statistical results for number of visible surface metastatic lesions in mice (n = 5 per group) receiving injection of the indicated cells by tail vein. *P < 0.05. (D) On day 35, mice were anaesthetized and tumours were collected. Representative bright‐field images of the tumours are shown. (E) The indicated cells (3 × 10^6^) were injected subcutaneously in nude mice. The tumour volume was measured on the indicated days. (F) When the mice were anaesthetized, the tumours were removed and weighed. *P < 0.05

Given that DUXAP9‐206 was associated with T classification, LN metastasis and poor prognosis of NSCLC, we further examined whether DUXAP9‐206 played a role in NSCLC proliferation and metastasis. For this purpose, we first used A549 cell lines to establish cells with stable overexpression or knockdown of DUXAP9‐206 labeled with firefly luciferase for injection of nude mice. As shown by luciferase live‐cell imaging and picric acid staining of metastatic lesions, mice bearing A549/DUXAP9‐206 cells enhanced pulmonary colonization. In contrast, DUXAP9‐206‐silenced cells inhibited pulmonary colonization (Figure 3A‐C). In addition, the results of survival analysis indicate that higher levels of DUXAP9‐206 expression are correlated with shorter survival time, while lower levels of DUXAP9‐206 expression show the opposite results (Figure S4).

Next, we assessed the effects of DUXAP9‐206 on cell proliferation. Using an experimental mouse xenograft model, we found that DUXAP9‐206‐transduced cells showed faster tumour growth rates than vector‐control cells. Conversely, silencing of DUXAP9‐206 significantly suppressed tumour growth rates (Figure 3D‐F). Taken together, these data strongly suggest that DUXAP9‐206 may act as a driver molecule to promote NSCLC metastasis and proliferation.

### 3.4 DUXAP9‐206 directly interacts with Cbl‐b in the cytoplasm

**Figure 4:**
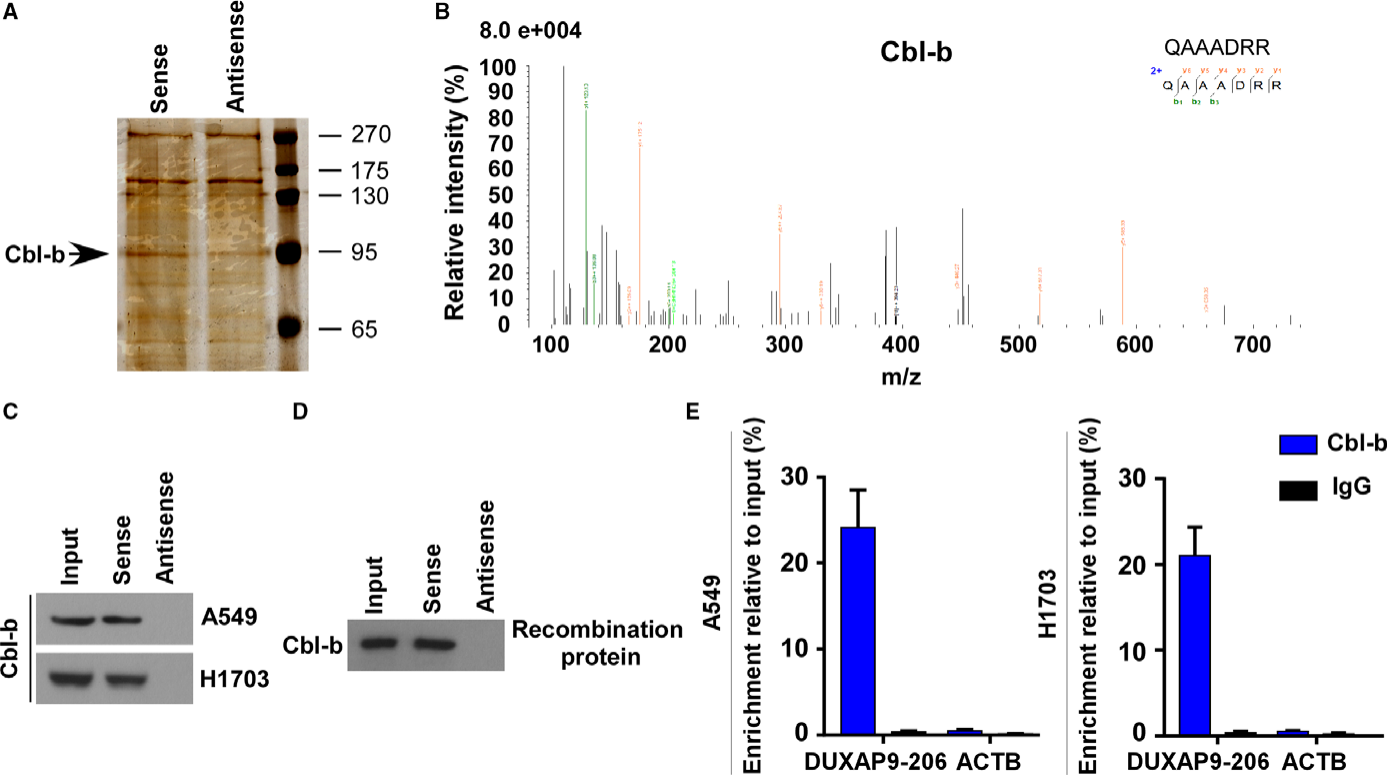
DUXAP9‐206 directly binds with Cbl‐b. (A) Imaging of RNA pull‐down experiment followed by silver staining. (B) Mass spectrometric analysis of Cbl‐b peptides. RNA pull‐down experiments with cell lysates (C) and recombinant proteins (D) of Cbl‐b to confirm the interaction of DUXAP9‐206 with Cbl‐b. (E) RIP assays show the association of DUXAP9‐206 and Cbl‐b

We next sought to explore the possible mechanism underlying the role of DUXAP9‐206 in regulating NSCLC progression in cytoplasmic processes. Previous studies have extensively shown that many lncRNAs may function by physically interacting with protein molecules and influencing cancer aggressiveness.^8^ To identify DUXAP9‐206‐associated proteins, we performed an RNA pull‐down assay followed by MS analysis. In our study, DUXAP9‐206 has been demonstrated to be mainly located in the cytoplasm of NSCLC cells (Figure 1H), which indicates that DUXAP9‐206 may function by interacting with other proteins mainly in cytoplasm. As a result, ten proteins identified from the results of MS analysis, with the highest abundance, located in the cytoplasm and involved in regulating tumour growth and metastasis were first analysed. These proteins included the serine/threonine‐protein kinase ULK2, stratifin (SFN), C‐X‐C chemokine receptor type 4 (CXCR4), sterile alpha motif domain‐containing protein 9 (SAMD9), tyrosine‐protein kinase (STYK1), tyrosine‐protein kinase Srms (SRMS), regulatory‐associated protein of mTOR (RPTOR), E3 ubiquitin‐protein ligase (Cbl‐b), T‐complex protein 1 subunit epsilon (CCT5) and serine/threonine‐protein kinase ULK4 (Figure S5). Using RNA‐Protein interaction prediction and RNA pull‐down analysis, we found that only Cbl‐b specifically associated with DUXAP9‐206 in the cytoplasm (Figure 4A‐C and Figure S6). In vitro RNA pull‐down with recombinant proteins of Cbl‐b revealed that DUXAP9‐206 indeed directly binds with Cbl‐b (Figure 4D). RNA immunoprecipitation (RIP) assays also showed enrichment of DUXAP9‐206 in complexes precipitated with antibody against Cbl‐b‐Flag compared with control IgG (Figure 4E). Thus, these results demonstrated that DUXAP9‐206 directly interacts with Cbl‐b in the cytoplasm.

### 3.5 DUXAP9‐206 binds with Cbl‐b to prevent degradation of EGFR

**Figure 5:**
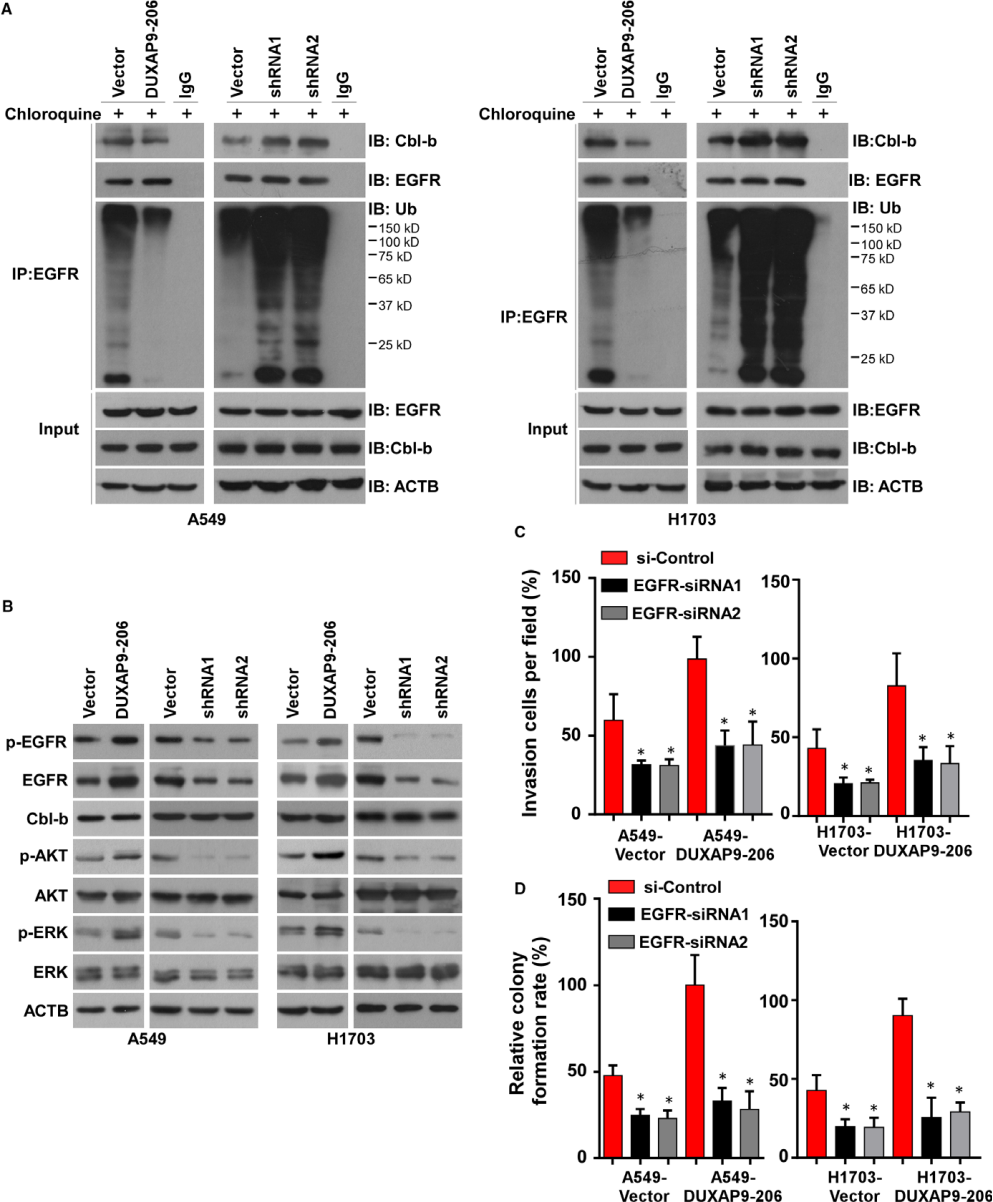
DUXAP9‐206 interacts with Cbl‐b to reduce the degradation of EGFR. (A) The interaction between EGFR and Cbl‐b with DUXAP9‐206 overexpression or knockdown was assessed by Co‐IP assays. (B) Western blotting analysis of the expression levels of Cbl‐b and the EGFR signaling pathway downstream molecules p‐AKT, AKT, p‐ERK and ERK with ectopic expression or silencing of DUXAP9‐206. Quantification of invading cells (C) and colony formation ability (D) in vector‐ and DUXAP9‐206‐overexpressing cells by silencing of EGFR

Cbl‐b, a member of the Cbl protein family, functions as an E3 ubiquitin‐protein ligase, which binds with activated tyrosine kinases, such as the EGFR, for lysosomal degradation leading to cellular responses including cell proliferation, motility/migration and invasion.^18^, ^19^ Combining the above results, it implicates a possibility that DUXAP9‐206 may promote NSCLC progression by directly binding with Cbl‐b to reduce the degradation of EGFR. To test this, we performed Co‐IP assays in cells with stable overexpression or knockdown of DUXAP9‐206 pretreated with the lysosome inhibitor chloroquine followed by EGF (100 ng/mL) treatment for 30 minutes. As shown in Figure 5A, overexpression of DUXAP9‐206 reduced the binding of Cbl‐b to EGFR and consequently reduced the ubiquitination of EGFR. However, knockdown of DUXAP9‐206 expression increased the association of Cbl‐b with EGFR and ubiquitination of EGFR (Figure 5A). These results indicated that DUXAP9‐206 inhibits the interaction between Cbl‐b and EGFR, then prevents the ubiquitination of EGFR.

We next investigated whether DUXAP9‐206 activated EGFR signaling. The results of Western blotting analysis showed that stable overexpression of DUXAP9‐206 increased the expression level of EGFR and downstream ERK/Akt activation, while knockdown of DUXAP9‐206 showed the opposite results (Figure 5B). Moreover, knockdown of Cbl‐b expression reversed the effects caused by DUXAP9‐206 overexpression (Figure S7A). To further verify the role of EGFR signaling in DUXAP9‐206‐induced cell invasion and proliferation, we analysed the impact by blocking EGFR signaling in DUXAP9‐206‐overexpressing cells. As shown in Figure 5C,D, overexpression of DUXAP9‐206 failed to rescue the invasive potential and colony formation capabilities in EGFR‐inhibition cells indicating that DUXAP9‐206 may exert biological effects through EGFR signaling. The inhibition of EGFR expression was confirmed by western blotting analysis (Figure S7B).

### 3.6 Clinical association of DUXAP9‐206 with EGFR signaling in NSCLC

**Figure 6:**
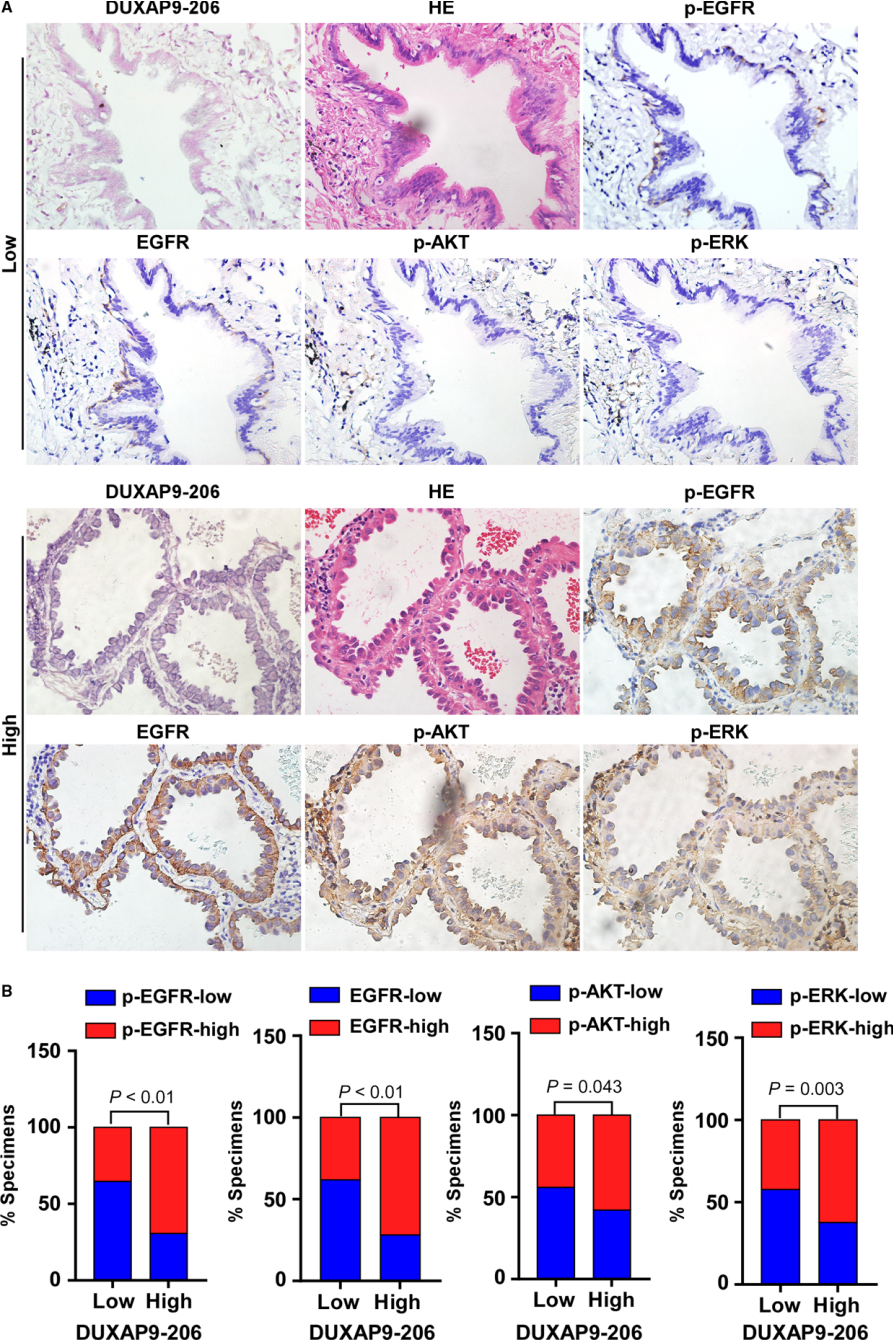
Clinical relevance of DUXAP9‐206 with EGFR, p‐EGFR, p‐AKT and p‐ERK in clinical specimens. (A) ISH analysis of DUXAP9‐206 and immunohistochemical analysis of p‐EGFR, EGFR, p‐AKT and p‐ERK expression in NSCLC tumour specimens. Sections were H&E stained to visualize the tumour structure and boundaries. Original magnification, ×400. (B) DUXAP9‐206 expression was positively associated with expression levels of p‐EGFR, EGFR, p‐AKT and p‐ERK in NSCLC specimens

To further investigate the clinical relevance of the above findings in NSCLC, we divided the expression of DUXAP9‐206 into high and low subgroups in 216 human NSCLC clinical specimens. As shown in Figure 6A,B, 64.7% (66 cases), 61.8% (63 cases), 55.9% (57 cases) and 57.8% (59 cases) of the samples with low DUXAP9‐206 expression (102 cases) exhibited low levels of p‐EGFR, EGFR, p‐AKT and p‐ERK respectively, whereas 69.3% (79 cases), 71.9% (82 cases), 57.9% (66 cases) and 62.3% (71 cases) of the samples with high DUXAP9‐206 expression (114 cases) showed high expression of p‐EGFR, EGFR, p‐AKT and p‐ERK respectively (*P* < 0.05). Collectedly, our data demonstrated that the expression of DUXAP9‐206 in NSCLC was positively associated with the expression of EGFR, p‐AKT and p‐ERK.

## 4 DISCUSSION

**Figure 7:**
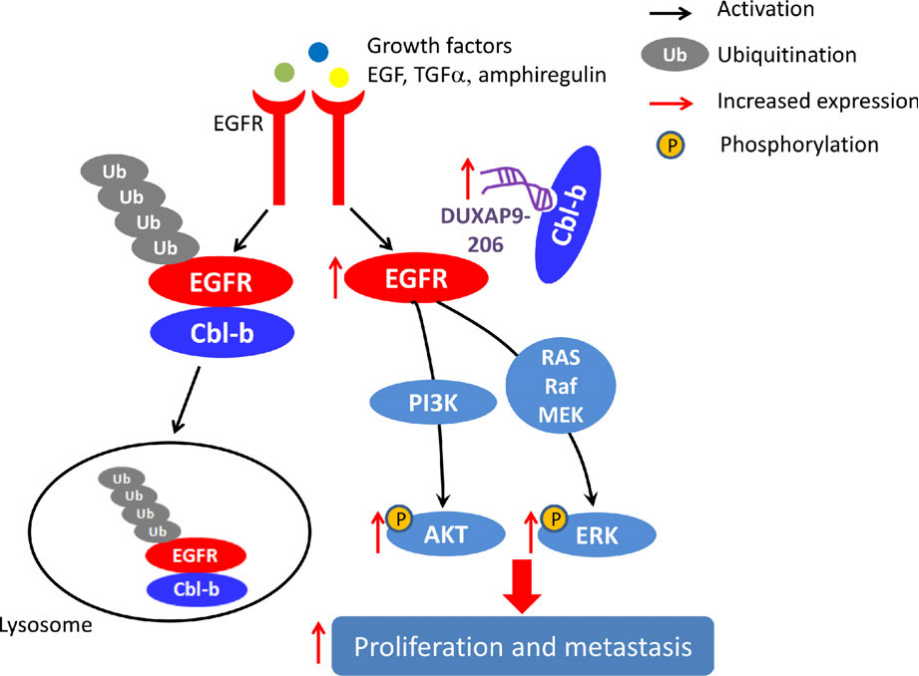
Proposed functional action of DUXAP9‐206 in modulating NSCLC proliferation and metastasis. LncRNA DUXAP9‐206 directly binds with Cbl‐b to augment EGFR signaling and promotes non‐small cell lung cancer progression

In the present study, we showed that lncRNA DUXAP9‐206 is significantly overexpressed in and highly related to clinical features and prognosis of NSCLC patients. LncRNA DUXAP9‐206 contributes to the proliferation and metastasis of NSCLC by binding with Cbl‐b to prevent the degradation of EGFR and thus ensures the activation of EGFR signaling (Figure 7).

Previous studies indicated that the progression of NSCLC is a complex and multifactorial event with multiple genetic changes. Identifying genes that contribute to the development of effective approaches to diagnose and treat NSCLC would be of great significance. In the present study, our results for the first time demonstrated that the lncRNA DUXAP9‐206, which is closely correlated with clinical NSCLC patient outcomes is a vitally important lncRNA that promoted the progression in NSCLC. Through RNA pull‐down and RIP assays, we found that DUXAP9‐206 directly interacted with Cbl‐b, an E3 ubiquitin ligase decreased the degradation of EGFR and thereby activated the EGFR signaling pathway. This conclusion is further supported by three lines of experimental evidence: first, DUXAP9‐206 directly binds with Cbl‐b. Second, the association of DUXAP9‐206 and Cbl‐b was indeed promoted in DUXAP9‐206‐overexpressing cells and abrogated in cells with DUXAP9‐206 knockdown. Third, overexpressing DUXAP9‐206 decreased the degradation of EGFR and increased the activation of EGFR signaling, whereas silencing of DUXAP9‐206 reversed these effects.

It has been well documented that ubiquitination and lysosomal degradation of EGFR requires direct binding with Cbl‐b. EGFR is activated by binding with the ligands EGF, TGFα or HB‐EGF and then, the EGFR‐ligand complexes may be internalized from the plasma membrane to the endosomes and ubiquitinated EGFR is efficiently targeted to lysosomes for degradation.^20^, ^21^ The recruitment of an E3 ubiquitin ligase, Cbl‐b, to activated EGFR is a key link that promotes its ubiquitination.^22^, ^23^ Cbl‐b, a member of the Cbl proteins, which include c‐Cbl, Cbl‐b and Cbl‐3 functions as a ubiquitin protein ligase (E3) for activated tyrosine kinases, such as EGFR and targets them for degradation.^24^, ^25^ Cbl‐b‐mediated EGFR degradation requires direct binding to the activated EGFR.^26^, ^27^, ^28^ Finally, EGFR signaling‐regulated biological effects, such as tumourigenesis and metastasis are attenuated via ubiquitination upon Cbl‐b.^29^ A previous study by Vennin et al demonstrated that the lncRNA H19, a precursor of miR‐675, directly binds to c‐Cbl and Cbl‐b mRNA through miR‐675 and increases the stability of EGFR and c‐Met in breast cancer.^30^ Jiang et al demonstrated that the lncRNA lnc‐EGFR specifically bound to EGFR and blocked its ubiquitination by c‐Cbl, a member of the Cbl family.^31^ In the present study, we found that the expression of Cbl‐b did not change when DUXAP9‐206 was overexpressed, but the interaction of DUXAP9‐206 and Cbl‐b was increased leading to a decrease in the binding of Cbl‐b to EGFR and consequently reducing the degradation of EGFR. Of note, this is the first report to identify an lncRNA that is directly involved in regulating the association of Cbl‐b and EGFR, further enriching and improving the degradation mechanism of EGFR. According to previous reports, Cbl proteins contain several highly conserved domains including an N‐terminal tyrosine kinase binding (TKB) domain and a RING finger. Cbl‐b‐mediated EGFR degradation requires the binding of the TKB domain to the activated EGFR.^29^ It is of interest to further explore whether DUXAP9‐206 also binds with the TKB domain of Cbl‐b, thus preventing the interaction of the TKB domain of Cbl‐b with EGFR. In addition, previous studies have reported that Cbl‐b can regulate a cohort of target genes involved in different signaling pathways.^32^, ^33^ Whether the oncogenic role of DUXAP9‐206 also requires additional mechanisms remains to be clarified in future studies. Furthermore, the clinical relevance of Cbl‐b inhibitors currently in development suggests that DUXAP9‐206 may indeed be a biomarker for response in NSCLC. It is of interest to further explore the role of these inhibitors in our presented models to determine if this is true. Taken together, our findings may further expand our understanding of the degradation process of EGFR in NSCLC and lay the foundation for identification of new therapeutic targets in the future.

Majority of the NSCLC patients worldwide are EGFR wild type.^34^ However, numerous receptor tyrosine kinase inhibitors target mutation‐positive NSCLC^35^, ^36^ and wild type EGFR patients lack effective treatments that significantly improve their survival and prognosis.^13^, ^34^ In this study, DUXAP9‐206 was found to inhibit the degradation of EGFR in EGFR wild type NSCLC cells, such as A549 and H1703, indicating that DUXAP9‐206 may be a promising therapeutic target for EGFR wild type patients in NSCLC. To further validate this result, we will collect wild type EGFR specimens from patients with NSCLC to compare the expression of DUXAP9‐206 with normal patient specimens, verify their relationship with relevant signaling pathways and analyse the correlation with survival and prognosis of patients. Whether this mechanism also plays an important regulatory role in the EGFR mutant patients should be investigated further.

In summary, our study indicates that DUXAP9‐206 and its regulated pathway is crucial for NSCLC and targeting DUXAP9‐206 may be pivotal in the diagnosis or treatment of NSCLC.

## CONFLICT OF INTEREST

The authors declare that they have no conflicts of interest.

## AUTHOR CONTRIBUTION

ZH, JW, TZ and SA designed experiments; TZ, SA, MC, JZ, SW, SL, BL, ZY, XZ, performed the experiments; TZ and SA analysed the data; ZH, JW, TZ and SA wrote the manuscript. All authors approved the final manuscript.

## References

[1] Siegel RL , Miller KD , Jemal A . Cancer statistics, 2017. CA Cancer J Clin. 2017;67:7‐30. 28055103 10.3322/caac.21387

[2] Ferlay J , Soerjomataram I , Dikshit R , et al. Cancer incidence and mortality worldwide: sources, methods and major patterns in GLOBOCAN 2012. Int J Cancer. 2015;136:E359‐E386. 25220842 10.1002/ijc.29210

[3] Hiley CT , Le Quesne J , Santis G , et al. Challenges in molecular testing in non‐small‐cell lung cancer patients with advanced disease. Lancet. 2016;388:1002‐1011. 27598680 10.1016/S0140-6736(16)31340-X

[4] ENCODE Project Consortium , Birney E , Stamatoyannopoulos JA , et al. Identification and analysis of functional elements in 1% of the human genome by the ENCODE pilot project. Nature. 2007;447:799‐816. 17571346 10.1038/nature05874 PMC2212820

[5] Bhan A , Soleimani M , Mandal SS . Long noncoding RNA and cancer: a new paradigm. Can Res. 2017;77:3965‐3981. 10.1158/0008-5472.CAN-16-2634 PMC8330958 28701486

[6] Batista PJ , Chang HY . Long noncoding RNAs: cellular address codes in development and disease. Cell. 2013;152:1298‐1307. 23498938 10.1016/j.cell.2013.02.012 PMC3651923

[7] Schmitt AM , Chang HY . Long noncoding RNAs in cancer pathways. Cancer Cell. 2016;29:452‐463. 27070700 10.1016/j.ccell.2016.03.010 PMC4831138

[8] Sun TT , He J , Liang Q , et al. LncRNA GClnc1 promotes gastric carcinogenesis and may act as a modular scaffold of WDR5 and KAT2A complexes to specify the histone modification pattern. Cancer Discov. 2016;6:784‐801. 27147598 10.1158/2159-8290.CD-15-0921

[9] Li C , Wang S , Xing Z , et al. A ROR1‐HER3‐lncRNA signalling axis modulates the Hippo‐YAP pathway to regulate bone metastasis. Nat Cell Biol. 2017;19:106‐119. 28114269 10.1038/ncb3464 PMC5336186

[10] Huang G , Jiang H , Lin Y , et al. lncAKHE enhances cell growth and migration in hepatocellular carcinoma via activation of NOTCH2 signaling. Cell Death Dis. 2018;9:487. 29706630 10.1038/s41419-018-0554-5 PMC5924759

[11] Blume‐Jensen P , Hunter T . Oncogenic kinase signalling. Nature. 2001;411:355‐365. 11357143 10.1038/35077225

[12] Gazdar AF . Epidermal growth factor receptor inhibition in lung cancer: the evolving role of individualized therapy. Cancer Metastasis Rev. 2010;29:37‐48. 20127143 10.1007/s10555-010-9201-z PMC3387977

[13] Laurie SA , Goss GD . Role of epidermal growth factor receptor inhibitors in epidermal growth factor receptor wild‐type non‐small‐cell lung cancer. J Clin Oncol. 2013;31:1061‐1069. 23401452 10.1200/JCO.2012.43.4522

[14] Pecze L , Blum W , Schwaller B . Routes of Ca^2+^ shuttling during Ca^2+^ oscillations: focus on the role of mitochondrial Ca^2+^ handling and cytosolic Ca^2+^ buffers. J Biol Chem. 2015;290:28214‐28230. 26396196 10.1074/jbc.M115.663179 PMC4653679

[15] Liu L , Guan H , Li Y , et al. Astrocyte elevated gene 1 interacts with acetyltransferase p300 and c‐Jun to promote tumor aggressiveness. Mol Cell Biol. 2017;37:e00456‐16. 27956703 10.1128/MCB.00456-16 PMC5311247

[16] Guan H , Song L , Cai J , et al. Sphingosine kinase 1 regulates the Akt/FOXO3a/Bim pathway and contributes to apoptosis resistance in glioma cells. PLoS ONE. 2011;6:e19946. 21625639 10.1371/journal.pone.0019946 PMC3097221

[17] Jiang L , Lin C , Song L , et al. MicroRNA‐30e* promotes human glioma cell invasiveness in an orthotopic xenotransplantation model by disrupting the NF‐kappaB/IkappaBalpha negative feedback loop. J Clin Invest. 2012;122:33‐47. 22156201 10.1172/JCI58849 PMC3248293

[18] Waterman H , Alroy I , Strano S , et al. The C‐terminus of the kinase‐defective neuregulin receptor ErbB‐3 confers mitogenic superiority and dictates endocytic routing. EMBO J. 1999;18:3348‐3358. 10369675 10.1093/emboj/18.12.3348 PMC1171415

[19] Lemmon MA , Schlessinger J . Cell signaling by receptor tyrosine kinases. Cell. 2010;141:1117‐1134. 20602996 10.1016/j.cell.2010.06.011 PMC2914105

[20] Sorkin A , von Zastrow M . Endocytosis and signalling: intertwining molecular networks. Nat Rev Mol Cell Biol. 2009;10:609‐622. 19696798 10.1038/nrm2748 PMC2895425

[21] Marmor MD , Yarden Y . Role of protein ubiquitylation in regulating endocytosis of receptor tyrosine kinases. Oncogene. 2004;23:2057‐2070. 15021893 10.1038/sj.onc.1207390

[22] Levkowitz G , Waterman H , Zamir E , et al. c‐Cbl/Sli‐1 regulates endocytic sorting and ubiquitination of the epidermal growth factor receptor. Genes Dev. 1998;12:3663‐3674. 9851973 10.1101/gad.12.23.3663 PMC317257

[23] Levkowitz G , Waterman H , Ettenberg SA , et al. Ubiquitin ligase activity and tyrosine phosphorylation underlie suppression of growth factor signaling by c‐Cbl/Sli‐1. Mol Cell. 1999;4:1029‐1040. 10635327 10.1016/s1097-2765(00)80231-2

[24] Blake TJ , Shapiro M , Morse HC 3rd , et al. The sequences of the human and mouse c‐cbl proto‐oncogenes show v‐cbl was generated by a large truncation encompassing a proline‐rich domain and a leucine zipper‐like motif. Oncogene. 1991;6:653‐657. 2030914

[25] Keane MM , Rivero‐Lezcano OM , Mitchell JA , et al. Cloning and characterization of cbl‐b: a SH3 binding protein with homology to the c‐cbl proto‐oncogene. Oncogene. 1995;10:2367‐2377. 7784085

[26] Meng W , Sawasdikosol S , Burakoff SJ , et al. Structure of the amino‐terminal domain of Cbl complexed to its binding site on ZAP‐70 kinase. Nature. 1999;398:84‐90. 10078535 10.1038/18050

[27] Kim M , Tezuka T , Suziki Y , et al. Molecular cloning and characterization of a novel cbl‐family gene, cbl‐c. Gene. 1999;239:145‐154. 10571044 10.1016/s0378-1119(99)00356-x

[28] Thien CB , Langdon WY . c‐Cbl: a regulator of T cell receptor‐mediated signalling. Immunol Cell Biol. 1998;76:473‐482. 9797470 10.1046/j.1440-1711.1998.00768.x

[29] Thien CB , Langdon WY . Cbl: many adaptations to regulate protein tyrosine kinases. Nat Rev Mol Cell Biol. 2001;2:294‐307. 11283727 10.1038/35067100

[30] Vennin C , Spruyt N , Dahmani F , et al. H19 non coding RNA‐derived miR‐675 enhances tumorigenesis and metastasis of breast cancer cells by downregulating c‐Cbl and Cbl‐b. Oncotarget. 2015;6:29209‐29223. 26353930 10.18632/oncotarget.4976 PMC4745721

[31] Jiang R , Tang J , Chen Y , et al. The long noncoding RNA lnc‐EGFR stimulates T‐regulatory cells differentiation thus promoting hepatocellular carcinoma immune evasion. Nat Commun. 2017;8:15129. 28541302 10.1038/ncomms15129 PMC5529670

[32] Liyasova MS , Ma K , Lipkowitz S . Molecular pathways: cbl proteins in tumorigenesis and antitumor immunity‐opportunities for cancer treatment. Clin Cancer Res. 2015;21:1789‐1794. 25477533 10.1158/1078-0432.CCR-13-2490 PMC4401614

[33] Thien CB , Langdon WY . c‐Cbl and Cbl‐b ubiquitin ligases: substrate diversity and the negative regulation of signalling responses. Biochem J. 2005;391:153‐166. 16212556 10.1042/BJ20050892 PMC1276912

[34] Mok TS , Wu YL , Thongprasert S , et al. Gefitinib or carboplatin‐paclitaxel in pulmonary adenocarcinoma. N Engl J Med. 2009;361:947‐957. 19692680 10.1056/NEJMoa0810699

[35] Rosell R , Moran T , Queralt C , et al. Screening for epidermal growth factor receptor mutations in lung cancer. N Engl J Med. 2009;361:958‐967. 19692684 10.1056/NEJMoa0904554

[36] Reinersman JM , Johnson ML , Riely GJ , et al. Frequency of EGFR and KRAS mutations in lung adenocarcinomas in African Americans. J Thorac Oncol. 2011;6:28‐31. 21107288 10.1097/JTO.0b013e3181fb4fe2 PMC3337520

