#  

**DOI:** 10.1111/jcmm.17270

**Published:** 2022-04-05

In Ting Zhu et al,^1^ the images of A549‐Vector (Migration, left panel) group and A549‐shRNA1 (Migration) group in Figure 2B contain errors. The correct figure is shown below. The authors confirm all results and conclusions of this article remain unchanged.

**FIGURE 2:**
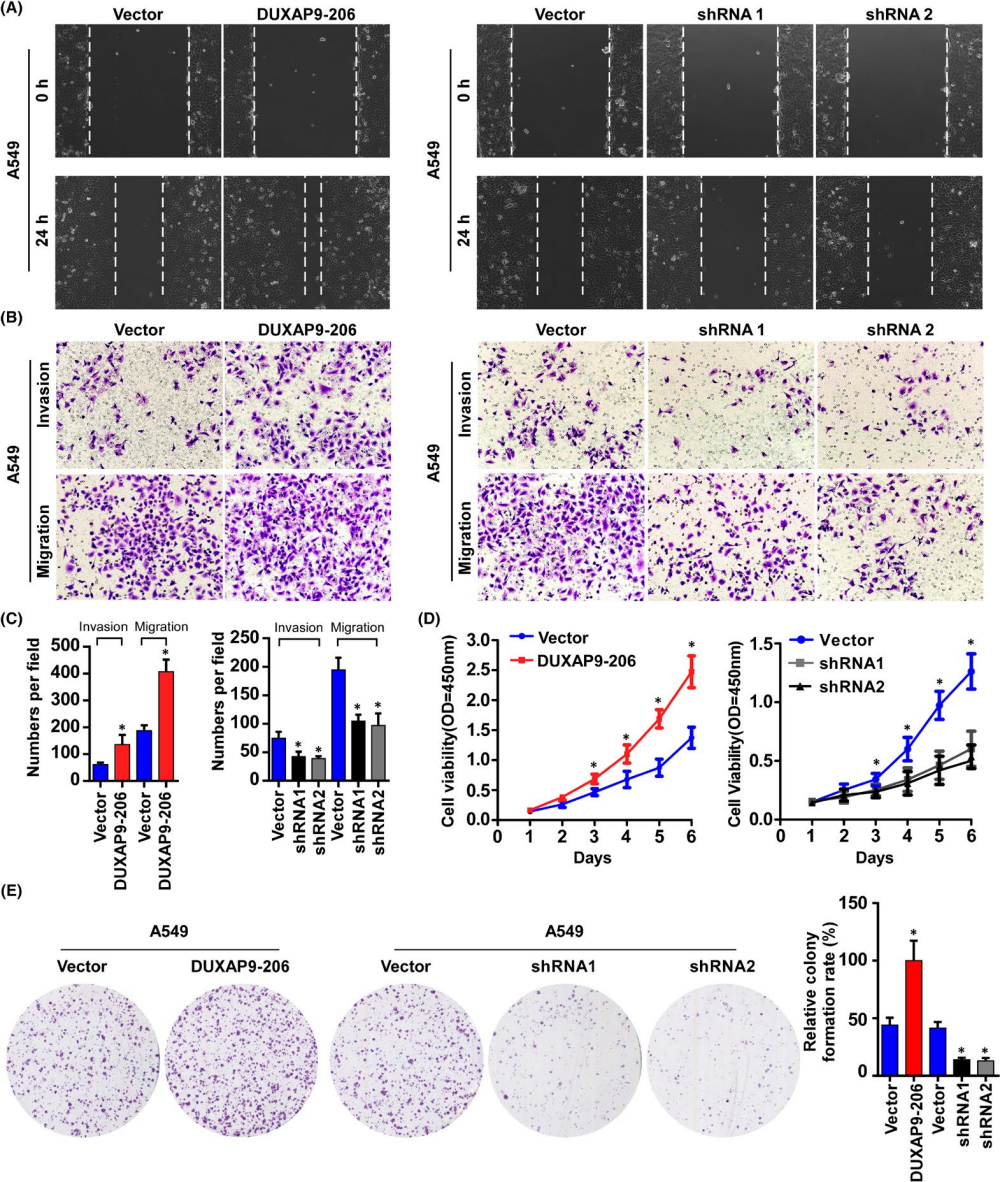
DUXAP9‐206 promotes NSCLC cell proliferation and invasion in vitro. (A) Representative micrographs of wound closures at 0 and 24 h after wounding. (B) The indicated invading or migrating cells analysed by Matrigel‐coated or noncoated Transwell assays, respectively. (C) Quantification of the indicated invading or migrating cells in 5 random fields analysed by Transwell assays. *p < 0.05. (D) MTT assays were performed in the indicated cells. (E) Representative micrographs (left panel) and quantification (right panel) of colony formation. *p < 0.05
