# Supplementary material for: LncRNA DUXAP9‐206 directly binds with Cbl‐b to augment EGFR signaling and promotes non‐small cell lung cancer progression
Source: J Cell Mol Med. 2018 Dec 4;23(3):1852–64. doi: 10.1111/jcmm.14085 (PMC6378200; doi:10.1111/jcmm.14085)
Supplement: Supplementary file 1 [file JCMM-23-1852-s001.doc]

**Supplementary Figures**

**
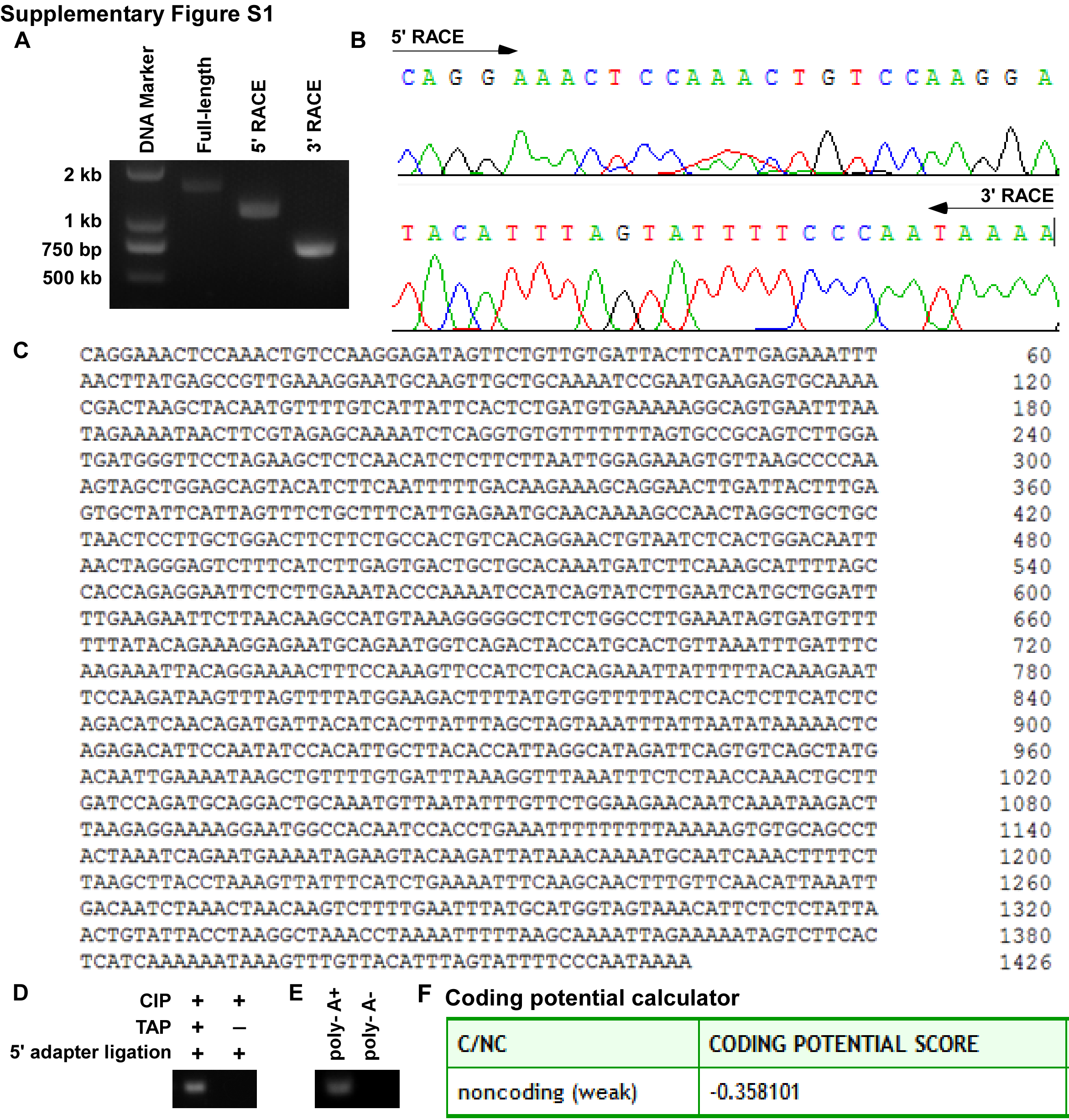
**

**Supplementary Figure S1. Characterization of DUXAP9-206.** Representative image **(A)** and sequencing **(B)** of PCR products from the 5'-RACE and 3'-RACE procedure. **(C)** The nucleotide sequence of full-length human DUXAP9-206. **(D)** DUXAP9-206 has a 5' cap structure. Total RNA was incubated sequentially with calf intestine alkaline phosphatase (CIP) and tobacco acid pyrophosphatase (TAP) to remove free 5'-P and the cap structure, respectively. Then, T4 RNA ligase was used to ligate an RNA adapter oligonucleotide and the RNA population (FirstChoice RLM-RACE Kit, Ambion). RT-PCR assay was used to detect DUXAP9-206. **(E)** RT-PCR analysis of the polyA+ RNA fraction and polyA- RNA fraction in the indicated cells. **(F)** A coding potential calculator was used to predict the coding potential of DUXAP9-206 (<http://cpc.cbi.pku.edu.cn/>).

**
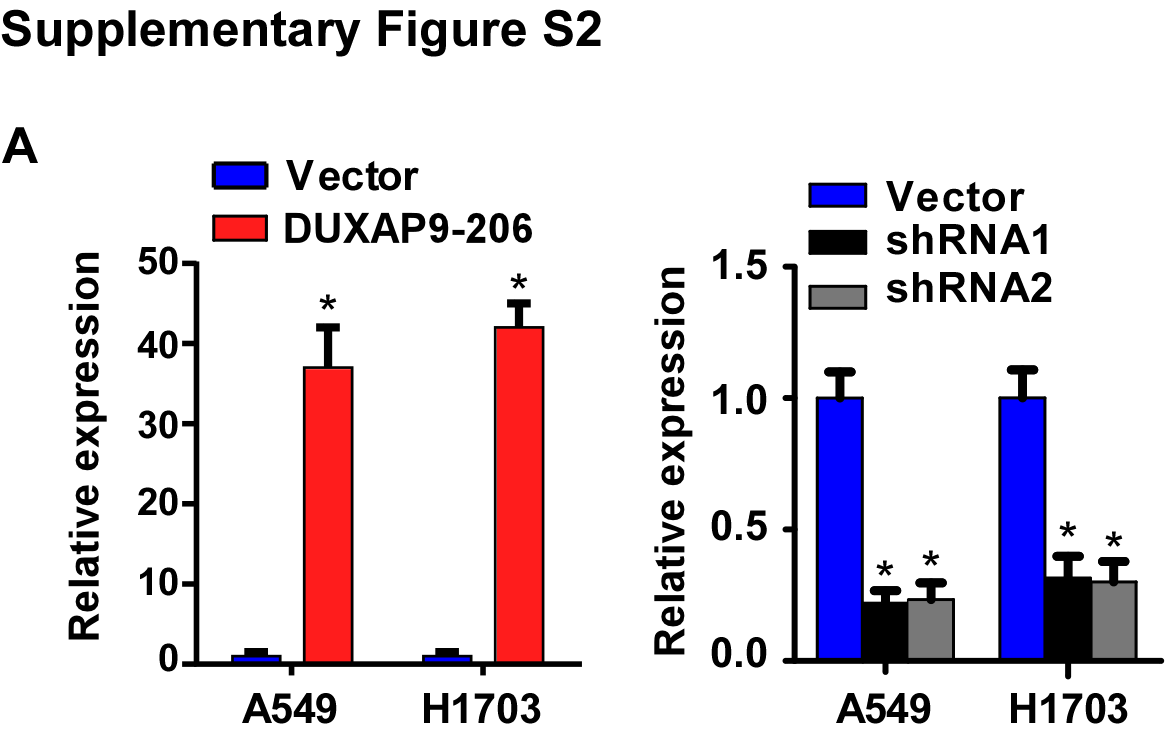
**

**Supplementary Figure S2.** Stable A549 and H1703 cell lines with overexpression **(A)** or knockdown **(B)** of DUXAP9-206 were established and verified by qRT-PCR analysis.

**
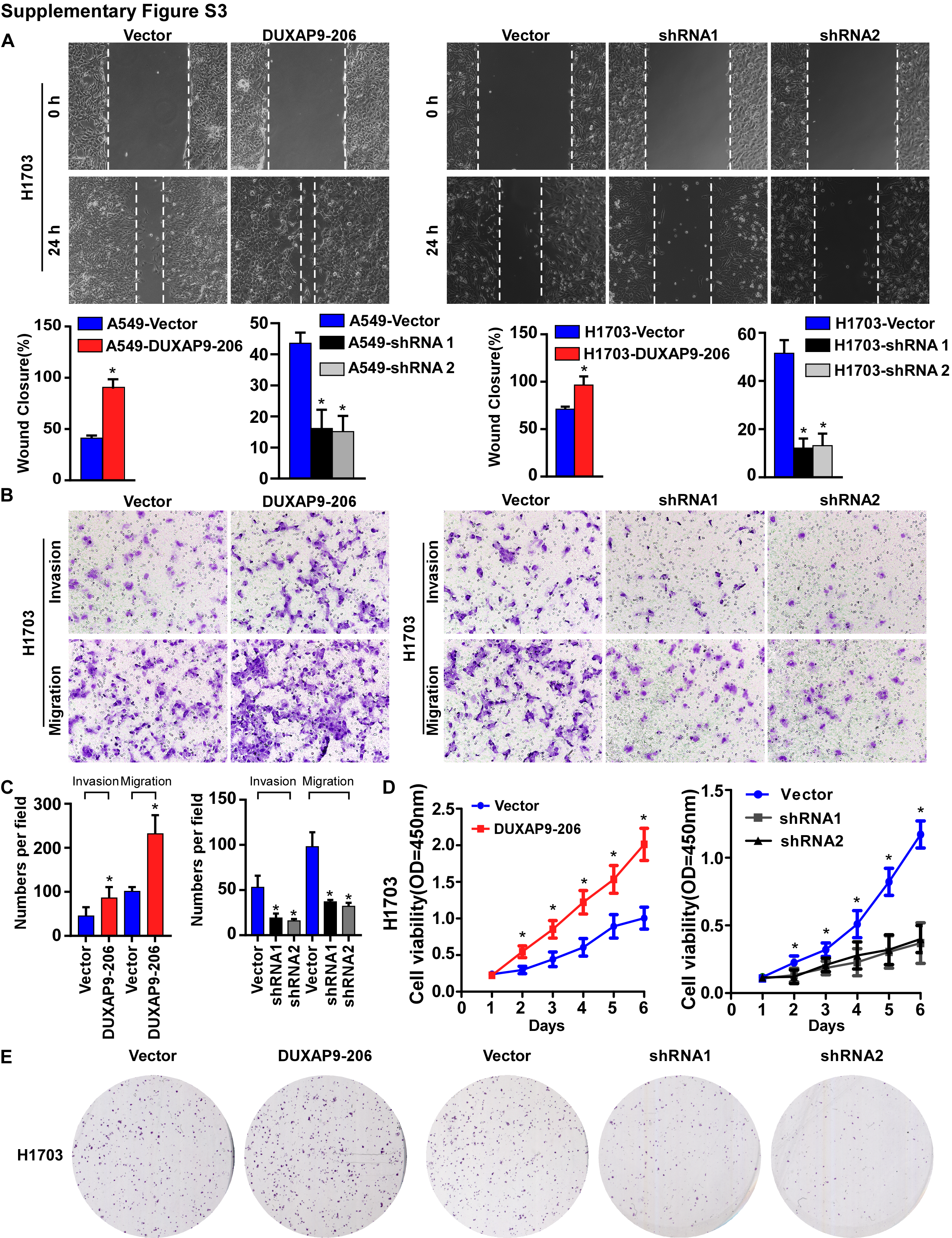
**

**Supplementary Figure S3.**  **DUXAP9-206 promotes invasion and proliferation in H1703 cells. (A)** Representative micrographs of wound closures after wounding. Quantification of the velocity in scratch assays. **P* < 0.05. **(B)** The indicated invading or migrating cells analyzed by Matrigel-coated or noncoated Transwell assays, respectively. **(C)** Quantification of the indicated invading or migrating cells analyzed by Transwell assays. **P* < 0.05. MTT assays (**D)** and colony formation assays (**E)** were performed in the indicated cells. **P* < 0.05.


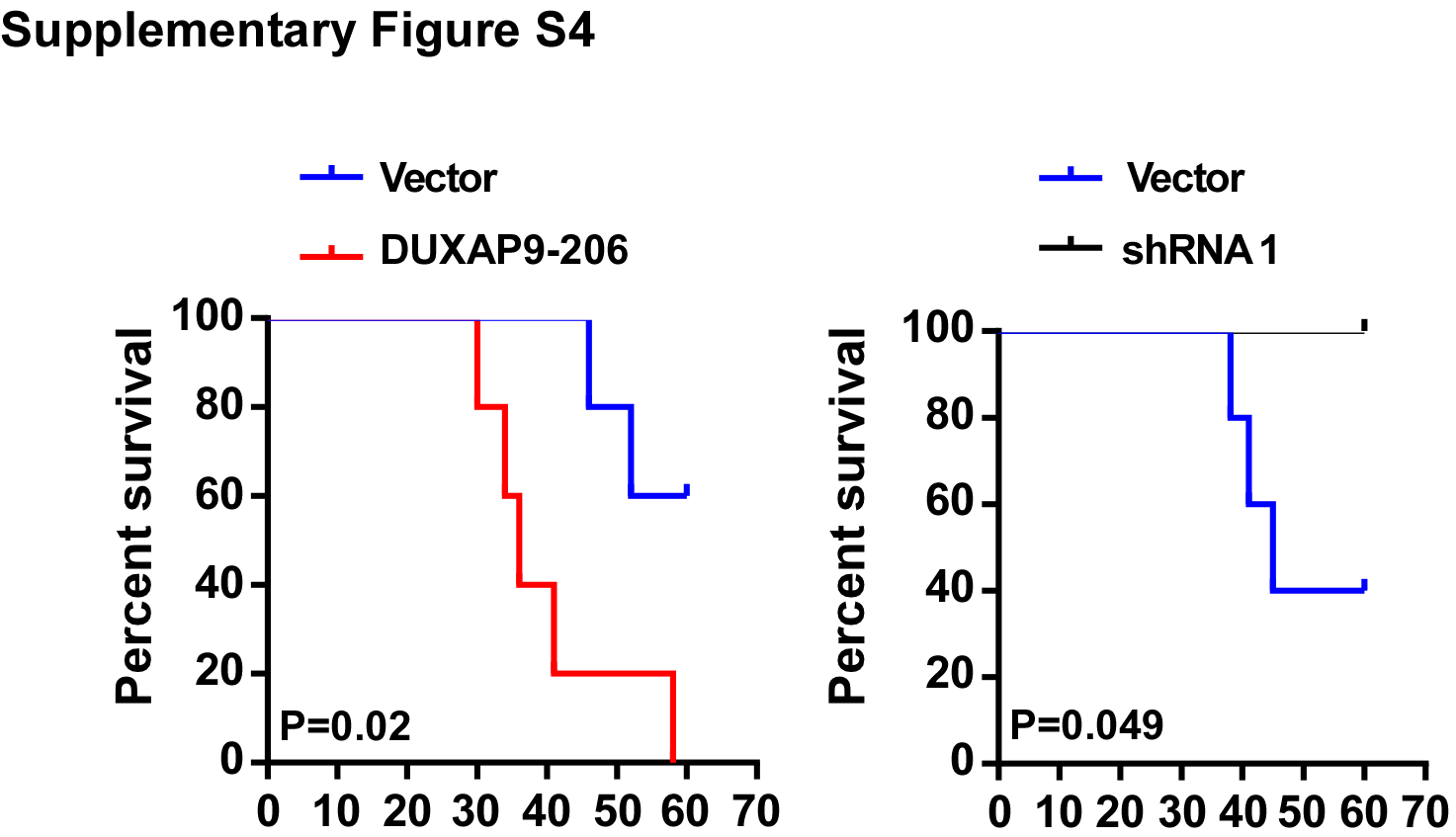


**Supplementary Figure S4.** Overall survival time of mice in indicated groups in the i.v. inoculation model (5 mice in each group).


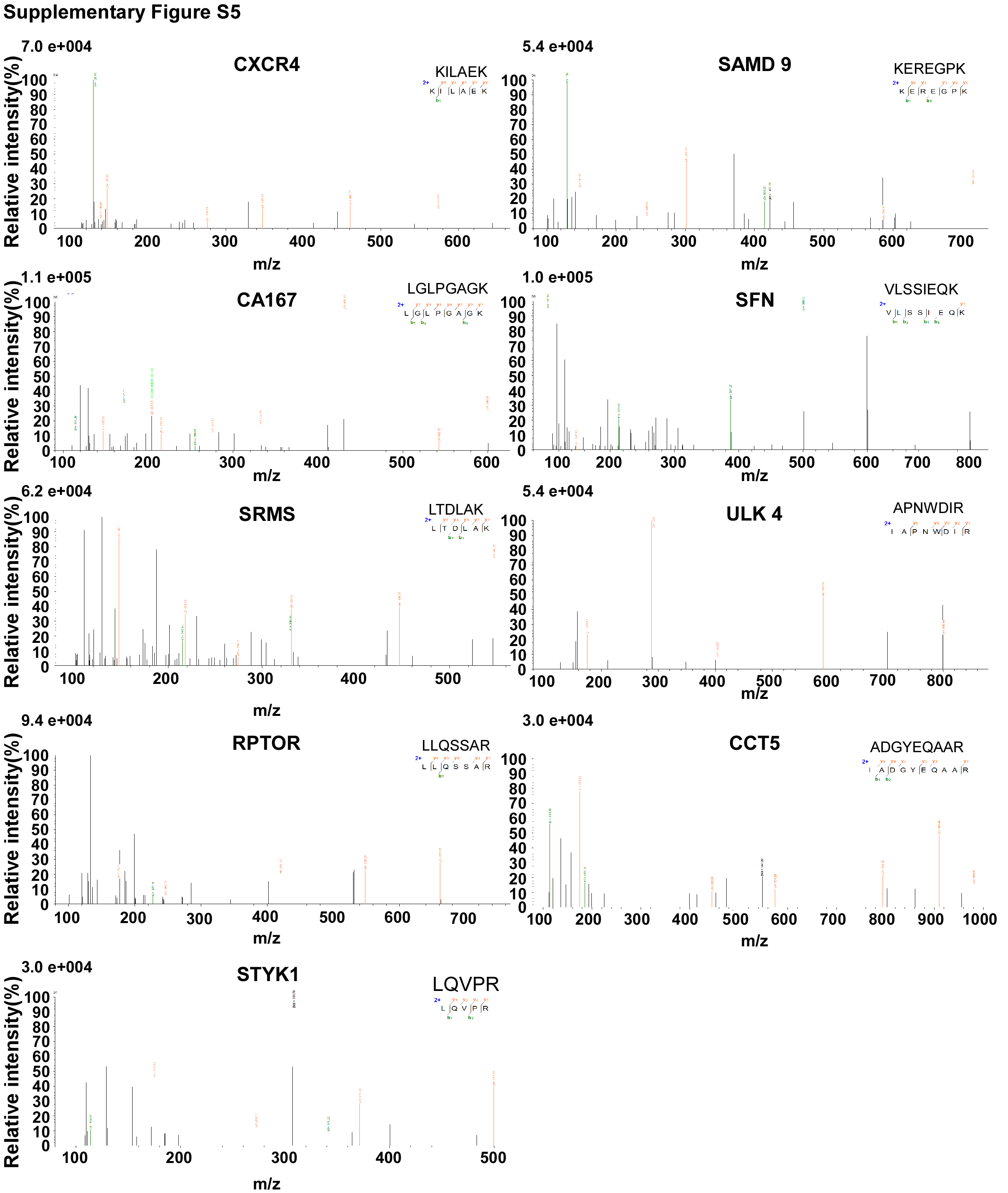


**Supplementary Figure S5.** **Annotated mass spectrum assigned to CXCR4, SAMD 9, CA 169, SFN, SRMS, ULK4, RPTOR, CCT5 and STYK1 peptides retrieved by DUXAP9-206.**


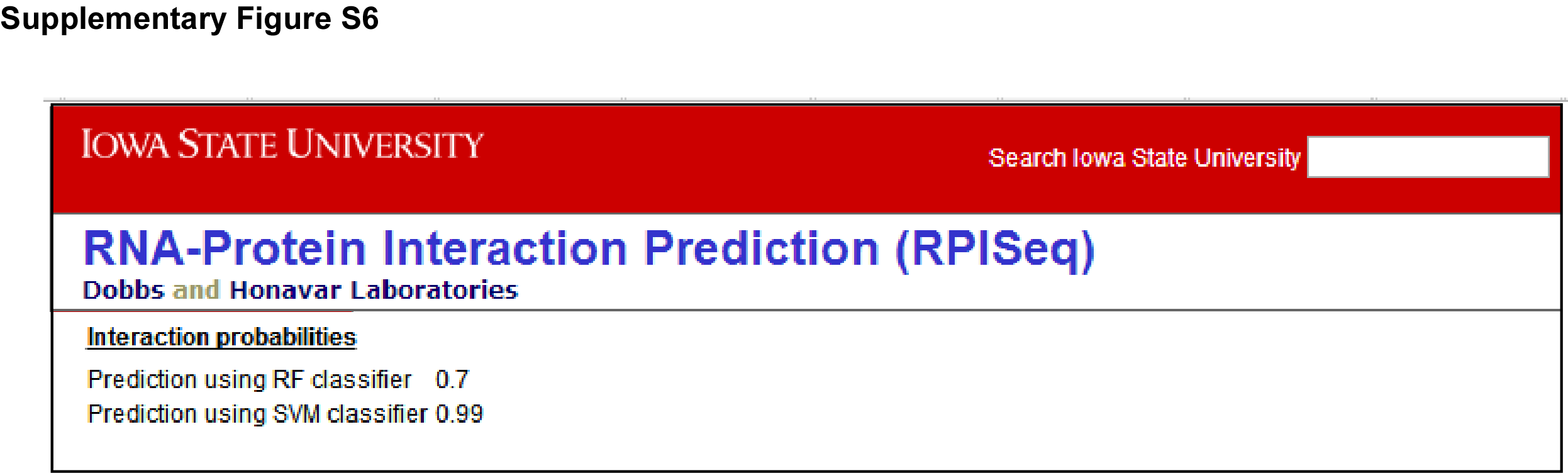


**Supplementary Figure S6. Prediction of the interaction between DUXAP9-206 and Cbl-b (**[**http://pridb.gdcb.iastate.edu/RPISeq/**](http://pridb.gdcb.iastate.edu/RPISeq/)**).**

**
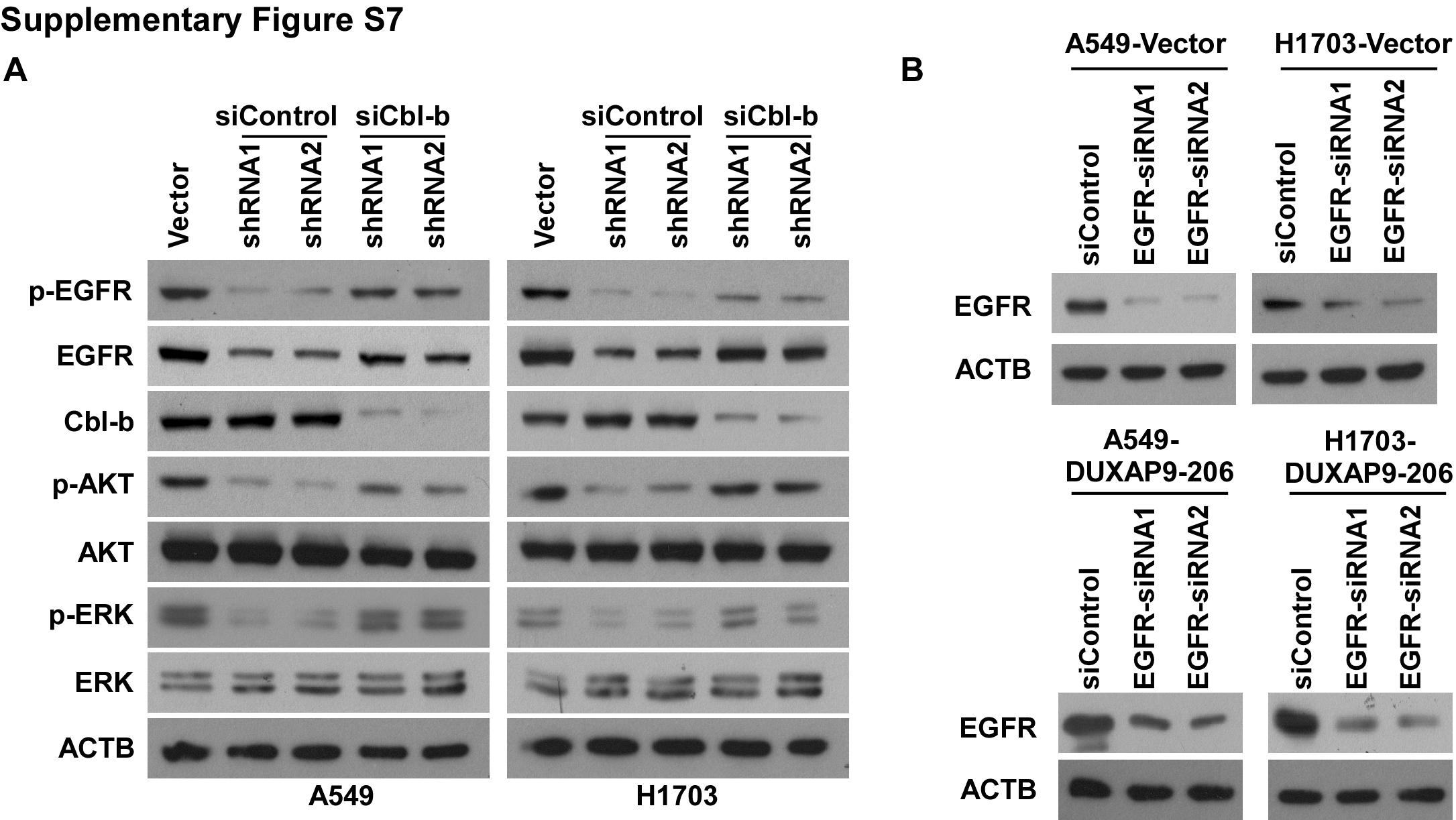
**

**Supplementary Figure S7. (A)** Cells with stable knockdown of DUXAP9-206 expression were transfected with Cbl-b siRNA or control siRNA respectively. Cell lysates were harvested and subjected for western blotting analysis of the expression levels of Cbl-b and the EGFR signaling pathway downstream molecules p-AKT, AKT, p-ERK, and ERK. **(B)** The expression of EGFR was determined by western blotting analysis in indicated cells.

**Supplementary Table 1. Clinicopathologic characteristics of NSCLC patients from SYSUCC cohort**

| **Characteristics** | **Number of cases** | **Percentage** |
| --- | --- | --- |
| **Gender** |  |  |
| Male | 161 | 74.5% |
| Female | 55 | 25.5% |
| **Age (years)** |  |  |
| ≥58 | 112 | 51.9% |
| ＜58 | 104 | 48.1% |
| **Clinical Stage** |  |  |
| I | 84 | 38.9% |
| II | 49 | 22.7% |
| III | 66 | 30.6% |
| IV | 17 | 7.9% |
| **T classification** |  |  |
| T1 | 36 | 16.7% |
| T2 | 114 | 52.8% |
| T3 | 54 | 25.0% |
| T4 | 12 | 5.6% |
| **LN metastasis** |  |  |
| Yes | 100 | 46.3% |
| No | 116 | 53.7% |
| **Distant metastasis** |  |  |
| Yes | 17 | 7.87% |
| No | 199 | 92.1% |

**Supplementary Table 2. Correlation between expressions of DUXAP9-206 and clinicopathologic features in NSCLC patients from SYSUCC cohort**

| **Characteristics** | **DUXAP9-206** | | | | **χ^2^** | | ***P*-value** | |
| --- | --- | --- | --- | --- | --- | --- | --- | --- |
|  | **Low** | | **High** | |  |  |  |  |
| **Gender** | |  | |  | |  | |  |
| Male | | 71 | | 90 | | 2.474 | | 0.116 |
| Female | | 31 | | 24 | |  |  |  |
| **Age (years)** | |  | |  | |  | |  |
| ≥58 | | 51 | | 61 | | 0.265 | | 0.606 |
| ＜58 | | 51 | | 53 | |  |  |  |
| **Clinical stage** | |  | |  | |  | |  |
| I | | 57 | | 27 | | 25.018 | | <0.01 |
| II | | 17 | | 32 | |  |  |  |
| III | | 20 | | 46 | |  |  |  |
| IV | | 8 | | 9 | |  |  |  |
| **T classification** | |  | |  | |  | |  |
| T1 | | 24 | | 12 | | 8.078 | | 0.044 |
| T2 | | 50 | | 64 | |  |  |  |
| T3 | | 21 | | 33 | |  |  |  |
| T4 | | 7 | | 5 | |  |  |  |
| **LN metastasis** | |  | |  | |  | |  |
| Yes | | 29 | | 71 | | 24.809 | | <0.01 |
| No | | 73 | | 43 | |  |  |  |
| **Distant metastasis** | |  | |  | |  | |  |
| Yes | | 8 | | 9 | | 0.000 | | 0.989 |
| No | | 94 | | 105 | |  |  |  |

**Supplementary Table 3. s**iRNA sequences

| **Target gene** | **Sequences (5’ to 3’)** |
| --- | --- |
| DUXAP9-206-siRNA1 | GGUCAGACUACCAUGCACUG |
| DUXAP9-206-siRNA2 | CACUCUUCAUCUCAGACAUC |
| EGFR-siRNA1 | GCAAAGUGUGUAACGGAAUAGGUAU |
| EGFR-siRNA2 | GGACUUCUUUCCCAAGGAA |
|  |  |

**Supplementary Table 4. Primers for the PCR** of the RACE analysis

| **Primer name** | **Sequences (5' to 3')** |
| --- | --- |
| 5' RACE | ACAGTGGCAGAAGAAGTCCAGCAAG |
| 3' RACE | ACAGAAAGGAGAATGCAGAATGGTC |

**Supplementary Table 5. Primer sequences for qRT-PCR**

| **Target gene** | **Sequences (5’ to 3’)** |
| --- | --- |
| DUXAP9-206 | Forward: CCAACTAGGCTGCTGCTAACTC |
|  | Reverse: CCTCTGGTGGCTAAAATGCTTTG |
| ACTB | Forward: GTTGTCGACGACGAGCG |
|  | Reverse: GCACAGAGCCTCGCCTT |
| U6 | Forward: CTCGCTTCGGCAGCACA |
|  | Reverse: AACGCTTCACGAATTTGCGT |
| GAPDH | Forward: GACTCATGACCACAGTCCATGC |
|  | Reverse: AGAGGCAGGGATGATGTTCTG |
|  |  |

**Supplementary Table 6. The sequence of the probe used for the in situ hybridization**

| **Probe name** | **Probe sequence (5' to 3')** |
| --- | --- |
| *In situ* hybridization | AGCTGACACTGAATCTATGCCT |
